# A Critical Review of Naphthalene Sources and Exposures Relevant to Indoor and Outdoor Air

**DOI:** 10.3390/ijerph7072903

**Published:** 2010-07-20

**Authors:** Chunrong Jia, Stuart Batterman

**Affiliations:** 1 School of Public Health, University of Memphis, 121 Browning Hall, Memphis, TN 38152 USA; E-Mail: cjia@memphis.edu; 2 Department of Environmental Health Sciences, University of Michigan, 1420 Washington Heights, Ann Arbor, MI 48109-2029 USA

**Keywords:** air quality, ambient air, exposure, indoor air, naphthalene, personal exposure, residences, risk, volatile organic compound

## Abstract

Both the recent classification of naphthalene as a possible human carcinogen and its ubiquitous presence motivate this critical review of naphthalene’s sources and exposures. We evaluate the environmental literature on naphthalene published since 1990, drawing on nearly 150 studies that report emissions and concentrations in indoor, outdoor and personal air. While naphthalene is both a volatile organic compound and a polycyclic aromatic hydrocarbon, concentrations and exposures are poorly characterized relative to many other pollutants. Most airborne emissions result from combustion, and key sources include industry, open burning, tailpipe emissions, and cigarettes. The second largest source is off-gassing, specifically from naphthalene’s use as a deodorizer, repellent and fumigant. In the U.S., naphthalene’s use as a moth repellant has been reduced in favor of para-dichlorobenzene, but extensive use continues in mothballs, which appears responsible for some of the highest indoor exposures, along with off-label uses. Among the studies judged to be representative, average concentrations ranged from 0.18 to 1.7 μg m^−3^ in non-smoker’s homes, and from 0.02 to 0.31 μg m^−3^ outdoors in urban areas. Personal exposures have been reported in only three European studies. Indoor sources are the major contributor to (non-occupational) exposure. While its central tendencies fall well below guideline levels relevant to acute health impacts, several studies have reported maximum concentrations exceeding 100 μg m^−3^, far above guideline levels. Using current but draft estimates of cancer risks, naphthalene is a major environmental risk driver, with typical individual risk levels in the 10^−4^ range, which is high and notable given that millions of individuals are exposed. Several factors influence indoor and outdoor concentrations, but the literature is inconsistent on their effects. Further investigation is needed to better characterize naphthalene’s sources and exposures, especially for indoor and personal measurements.

## 1. Introduction

Naphthalene is a toxic air pollutant widely found in ambient and indoor air due to emissions from the chemical and primary metals industries, biomass burning, gasoline and oil combustion, tobacco smoking, the use of mothballs, fumigants and deodorizers, and many other sources. Naphthalene is widely used as an intermediate in the production of phthalic anhydride (66,000 metric tons in the U.S. in 2000), surfactants (27,000 tons) and pesticides (14,000 tons) [1]. It is also found in many other environments, e.g., 40% of National Priority List (Superfund) sites contain naphthalene in soils [2]. Naphthalene is also called tar camphor, naphthene, naphthalin, naphthaline, mothballs, mothflakes and white tar; trade names include albocarbon, dezodorator, mighty 150, and mighty RD1 [2,3]. Naphthalene is rather a special compound in terms of its properties and chemical structure. It is a flammable white solid with the formula C_10_H_8_ and the structure of two fused benzene rings, with melting and boiling points of 80.5 and 218 °C, respectively. Its odor is fairly distinctive but not unpleasant, and its odor threshold is about 440 μg m^−3^ [2]. It is classified as a semi-volatile organic compound (SVOC) due to its vapor pressure of 0.087 mmHg at 25 °C, which is just below the 0.1 mmHg cut-off often used to define volatile organic compounds (VOCs) [4]. However, naphthalene sublimes rapidly at room temperatures. Due to its bicyclic aromatic structure, naphthalene is also a polycyclic aromatic hydrocarbon (PAH), and it is the most volatile member of this group. Naphthalene has been a target compound in environmental studies examining VOCs, SVOCs and PAHs. While known as a common and widespread air contaminant for many years [5], naphthalene received relatively little attention prior to the finding of its carcinogenicity in rats in 2000 [3].

The general public is exposed to naphthalene mainly through inhalation of ambient and indoor air, followed by dietary and non-dietary ingestion [6–8]. However, no study has explicitly compared exposures across multiple routes for the general population. One estimate of the average intake rate for inhalation is 19 μg day^−1^, and 0.002 to 4.0 μg day^−1^ for ingestion of water [9]. For nonsmokers exposed to environmental tobacco smoke (ETS) in their residences, the intake rate due to residential ETS is 1 to 3 μg day^−1^ [10]. High exposures can occur among workers in industries where naphthalene is present at high concentrations, e.g., mothball manufacturing and creosote-impregnation facilities [11]. High exposures also have been suggested among forest firefighters [12].

Exposure to naphthalene has been linked to a number of adverse health effects [2,13–16]. The major non-cancer endpoints are hyperplasia and metaplasia in respiratory and olfactory epithelium, respectively [17], and the cancer endpoint of concern are nasal tumors [16]. US EPA’s current risk assessment dates from 1998, but a 2004 draft report and peer review [18] are under consideration to update the inhalation cancer assessment, as well as the noncancer and oral risks, in response to a National Toxicology Program chronic inhalation study [3]. This draft report has incorporated new information and new risk assessment methods, with the effect of substantially lowering exposure limits. It is being revised to include cancer and noncancer effects for both oral and inhalation exposure, and is expected to be publicly available in mid 2011; a final assessment is expected in 2012. The science behind the toxicity of naphthalene is controversial, and industry stakeholders have organized a “Naphthalene Research Committee” with a 5 year (2007–2011) and reported $5.25 million program to further investigate toxicokinetics, including naphthalene’s mode of action and effects at low doses, and have sponsored a symposium and several review papers [5,19,20].

Naphthalene has been the subject of exposure and risk assessments since 1980 [5]. Table 1 summarizes U.S. guidelines for short- and long-term hazards and cancer risks. Occupational exposure limits and guidelines formulated for naphthalene include OSHA’s permissible exposure limit (PEL) and ACGIH’s threshold limit value (TLV), both 50 mg m^−3^ as a time weighted average (TWA, measured over an 8-hr period), which is much higher than levels usually encountered by the general public. Indoor and ambient standards have not been established. U.S. EPA lists naphthalene as a Hazardous Air Pollutant (HAP), as a Mobile Source Air Toxic (MSAT) for which regulations are to be developed under the U.S. Clean Air Act, and as one of 16 priority PAHs. U.S. EPA has set a chronic reference concentration (RfC) of 3 μg m^−3^, based on a chronic inhalation mouse study reporting nasal effects, included hyperplasia and metaplasia in respiratory and olfactory epithelium. This RfC includes an uncertainty factor of 3,000 [15]. In the same assessment, EPA stated that naphthalene is a possible human carcinogen, but that available data were inadequate to establish a causal association and thus no quantitative guidance was given. In the 2002 National-Scale Air Toxics Assessment, U.S. EPA [21] used a cancer unit risk estimate (URE) of 3.4 × 10^−5^ per μg m^−3^, which represents the upper-bound (95% confidence) excess lifetime cancer risk estimated to result from continuous exposure at a concentration of 1 μg m^−3^. This value was adopted from the California Office of Environmental Health Hazard Assessment [22]. Naphthalene’s carcinogenicity potential was increased 3-fold with a URE of 1 × 10^−4^ per μg m^−3^ in the draft revision to EPA’s risk assessment [18] and naphthalene was considered as a probable human carcinogen, based on increased risk of rare nasal tumors (respiratory epithelial adenomas and olfactory epithelial neuroblastomas in male rats) found in the NTP [3] study, although human evidence is lacking [23]. This draft URE value gives a concentration of 0.01 μg m^−3^ for chronic inhalation that corresponds to a cancer risk of 10^−6^. Several U.S. states utilize guideline values that are more comparable to the RfC and that also apply to ambient air. IARC [14] summarizes limit values for other countries. The World Health Organization also is considering the development of an indoor air guideline for naphthalene [24].

Meaningful estimates of exposures for the general public can only be developed with appropriate and adequate information on concentrations in the various microenvironments frequented by people. Often, the home residence is considered the most important microenvironment in exposure assessment since this is where people spend most of their time, e.g., 69% in the U.S. [25] and 66% in Canada [26]. Focusing on current exposures is important as these represent the starting point for risk reduction efforts, and since exposures to many pollutants have declined due to actions aimed at controlling pollutant use, emissions, and concentrations.

This paper reviews the more recent naphthalene literature, focusing on sources and concentrations in residential indoor environments and outdoor air. We derive representative estimates of naphthalene concentrations that can be used to estimate background levels and risks, and we discuss determinants of exposure, information intended to inform the development of policies and regulations aimed at improving air quality and reducing exposures. Our scope largely excludes occupational and industrial settings, though we note that many workers may be exposed, e.g., in an older study, NIOSH [27] estimated that 112,700 workers were potentially exposed to naphthalene. Exposure concentrations from 0.7 to 93.2 μg m^−3^ have been reported in industrial settings in Germany [28], and have exceeded 1,000 μg m^−3^ in aluminum, coke, creosote and iron industries in Europe [29], much higher than seen in the outdoor, indoor and personal air studies discussed in the body of this paper.

## 2. Methods

### 2.1. Literature sources

We searched the scientific literature dealing with sources, concentrations and/or exposure measurements of naphthalene in residences and ambient air. Five major online databases were used: (1) Science Citation Index Expanded (1900-present, ISI Web of Knowledge), which contains peer-reviewed journal articles; (2) Conference Proceedings Citation Index - Science (ISI Web of Knowledge), which searches in many conferences, symposia, seminars, colloquia, workshops, and conventions in a wide range of disciplines; (3) ScienceDirect, which contains recent and but still in-press peer-reviewed journal articles, many of which are not searchable in the ISI listings; (4) Medline (CSA), which focuses on biomedical literature but includes medical, epidemiological and exposure studies reporting on chemical exposures; and (5) ProQuest, which includes dissertations and theses that may not be published in the journal literature. We also reviewed proceedings, obtained on CD, from the three major international societies addressing exposure assessment and air quality: (1) Air and Waste Management Association Annual Conference & Exhibition (annual from 2000 to 2008); (2) Indoor Air - International Conference on Indoor Air Quality and Climate (1999 to 2008 held tri-annually); and (3) International Society of Exposure Science (2006 to 2008). We also searched books and internet resources, as well as the references cited in identified materials. An Endnote database was assembled.

### 2.2. Data analysis

To facilitate analyses, concentrations reported in units of ppb or ppm were converted to μg m^−3^ at standard conditions (25 °C, 1 atm), using the relationship of 1 ppb = 5.2 μg m^−3^.

We focused on measures of central tendency (medians and means) and extreme values (90th and 95th percentiles, maximum). Given a lognormal distribution, a common assumption in air pollution studies [36,37,38], the geometric mean (GM) can be derived as [37]:

(1) $$\text{GM}=\text{AM}/\sqrt{1+{\text{CV}}^{2}}$$

where AM = arithmetic mean; and CV = coefficient of variation = SD/AM where SD = arithmetic standard deviation. Arithmetic averages may be unduly influenced by extreme values. The median is a more robust measurement, and this statistic is emphasized. Because the GM and the median are approximately equal under a log-normality assumption, the median was approximated using the GM if the median was not reported.

Pollutant measurements can be influenced by many factors, e.g., community type (industrial, urban, suburban, rural and remote areas), season, and the presence of smoking. We derived representative ranges of average and median concentrations (minimum to maximum reported across the studies) by targeting non-smoking populations in urban areas; this was also the typical study design. Four measurement types were excluded in deriving representative concentration ranges: (1) concentrations collected in smoking residences, if explicitly stated and reported separately; (2) concentrations measured in rural and remote areas that are commonly treated as “background” levels; (3) “old” data, that is, samples collected before around 1990. Data prior to 1990 has been previously reviewed (as reported in Section 3.2.1), and sometimes showed high concentrations; and (4) data with known or suspected measurement bias, e.g., small sample size, and unreasonably high concentrations.

Data quality or representativeness issues are suspected or seem likely in those studies that used small sample sizes or in which measurements were below or near method detection limits (MDLs). Of course, all measurements are subject to various types of errors, e.g., sample losses and analysis issues, however, errors are especially likely in ambient settings where naphthalene concentrations are generally low. Studies in the peer-reviewed literature are emphasized.

## 3. Results

### 3.1. Emission sources of ambient and indoor naphthalene

Emission source of naphthalene may be classified in several ways. Based on the generation mechanism, naphthalene is emitted as a product of incomplete combustion, e.g., from wood, straw, tobacco, gasoline and diesel combustion, and from evaporation or sublimation of naphthalene-containing materials, e.g., coal tar, crude oil, petroleum products, moth repellents and air fresheners. Emissions can be classified as natural sources, e.g., wildfires, and as anthropogenic sources, which are a much larger and more significant source of exposure. From an emission inventory perspective, it is useful and common to classify sources as industrial, mobile, agricultural, natural and domestic [39]. In California, for example, gasoline engines, diesel engines, slow cure asphalt, consumer products, and industrial sources are estimated to contribute 44, 9, 13, 15, and 19% of the statewide total emissions, respectively [40]. Emission factors (EF) for selected sources are presented in Table 2. Environmental releases of naphthalene reported under the U.S. Toxics Release Inventory (TRI) in 2002 were 2.07 million pounds to air (mostly from combustion) and 0.37 million pounds to land [2]; this inventory emphasizes industrial sources. Considerably larger emissions have been reported due to naphthalene’s use as a moth repellent, e.g., 5,500 metric tons were estimated to have been released in 1989 from this use [61]; a more recent estimate of naphthalene’s use as a pesticide is 3,400 metric tons [62]. Both EFs and inventory estimates have many gaps, and several types of sources have particularly large uncertainties, as discussed below.

#### 3.1.1. Combustion sources

The formation of naphthalene (and other PAHs) during combustion has been extensively studied. Combustion is considered to be the single largest emission source of naphthalene [2]. Fuel type, oxygen supply and temperature are the major factors affecting emissions [63]. Naphthalene formed during combustion is predominantly in the vapor phase [64], e.g., 90% was measured from burning of rice and bean straw [58].

Emission factors (EF) for many biomass fuels have been determined in the laboratory under controlled conditions (Table 2). EFs for open fires have been comprehensively reviewed [65]. For biomass fuels, these factors span over three orders of magnitude: medians are 40, 17, and 8 mg kg^−1^ for wood, coal and crop straw, respectively. Open burning of agricultural residues is a significant source in developing countries. Burning of rice and bean straw in China, for example, emits an estimated 110 to 126 and 13 to 26 metric tons yr^−1^ of naphthalene, respectively [58]. Emissions from wildfires also can be significant. In China, wildfires release 141 metric tons yr^−1^, including 68% and 32% from forest and grassland fires, respectively [66]. In Africa, wildfire emissions are projected to exceed anthropogenic emissions [67]. Wildfire emissions can contribute to naphthalene levels in urban areas by long range transport [68,69]. Burning of wood and coal for domestic heating and cooking are additional sources. Overall, naphthalene EFs for biomass combustion vary considerably by region and time, and uncertainties are high.

Vehicle emissions represent an important naphthalene source in urban areas. For gasoline-powered cars and light-trucks, EFs for vehicles with and without catalytic converters are about 1 and 50 mg km^−1^, respectively [50]. For diesel-powered vehicles, EFs are about 10, 505, 276, and 20 mg km^−1^ in idle, creep, transient, and cruise modes, respectively [53]. Naphthalene has been found in exhausts of helicopters and ships [55–57].

Second-hand cigarette smoking is among the largest contributor to personal exposures of naphthalene. EFs of naphthalene in the side-stream smoke measured in the laboratory range from 12 to 15 μg cigarette^−1^ for commercial cigarettes [44], and slightly higher for research cigarettes [45]. Under realistic conditions, reported EFs range more broadly, from 17 to 54 μg cigarette^−1^ and depend on air exchange rates, furnishing level [47] and smoking condition [70]. All or most of these emissions are due to the side stream smoke [44,70]. Naphthalene EFs for cigarettes across these studies are reasonably consistent.

Mosquito coils may be an important source of naphthalene exposure. These coils are used both indoors and outdoors; in Asia, they are commonly used in sleeping areas during the night. A recent chamber study measured emissions from 4.8 to 19.5 μg h^−1^ from burning mosquito coils [71].

A final combustion source, also potentially important indoors, is the burning of incense sticks. While very common in Asian residences and temples, EF estimates are lacking.

#### 3.1.2. Pyrolysis sources

Naphthalene emissions during pyrolysis have been documented. The mean naphthalene concentration in the exhaust of flares from the pyrolysis of scrap tires is 150 (range of 0.11 to 543) μg m^−3^ [72]. An EF of 20.2 μg g^−1^ was determined for the pyrolysis of liquid crystal wastes [73].

Food cooking can involve pyrolysis when organic compounds are partially cracked to small fragments, which then recombine with radicals to form relatively stable PAHs. Frying and boiling yielded 0.25 to 4.4 μg m^−3^ of excess naphthalene concentrations per kg of fish or pork chops, while boiling generated only 0.028 to 0.045 μg m^−3^ kg^−1^ [74].

#### 3.1.3. Off-gassing and volatilization

Naphthalene is a natural component in coal tar and crude oil with typical contents of 11% and 1.3%, respectively [2]. It is also a constituent of gasoline, diesel and jet fuel. In gasoline, the naphthalene content has been expressed as 1.04 mg g^−1^ [50], 69 to 2600 mg L^−1^ [75], and 0.15 to 0.18% (w/w) [76]. Naphthalene’s content varies greatly by brand and grade, and “premium” gasoline tends to have higher concentrations than “regular” gasoline [75]. In diesel, the naphthalene content varies from 6.6 to 1,600 mg L^−1^ [75]. In jet fuel, the naphthalene content is 0.26% (w/w) [77].

The use of naphthalene as a moth repellent has been stated to be the second largest naphthalene exposure source after combustion [2], although its use in this application exceeds rates in the available emission inventories (described later). A variety of materials are used as “moth preventatives” in the form of balls, crystals, flakes, cakes, blocks, bars and “nuggets,” available loose and packaged in porous bags and boxes, clothes hangers, and other niceties, and sometimes scented with cedar, lavender and other fragrances. In the U.S., most of these products are made of essentially pure *p*-dichlorobenzene [78], which avoids the flammability hazard associated with naphthalene. However, naphthalene remains readily available, e.g., sold as “old fashioned mothballs” or flakes, except in California where it has not been registered under Proposition 65. At the federal level, naphthalene is registered for use on indoor sites as a moth repellant in the form of balls and flakes (it is not formed in blocks and other configurations). Based on industry reports [79], the U.S. moth preventative consumer sales totals $14 million annually, of which 41% can be identified as naphthalene products. However, these figures are incomplete, e.g., many merchandisers and bulk sales are excluded, and actual sales are likely multiples of this value. Also, naphthalene’s actual share will be lower since many retailers only stock items that can be sold nationally and, as mentioned, California sales for this purpose are not permitted.

In chambers, the emission rate from moth repellents made of essentially pure naphthalene is 0.16 to 0.19 mg g^−1^ h^−1^ [80], and it tends to last longer than *p*-dicholorobenezene in the same application. Clothes stored with moth repellents may adsorb naphthalene and subsequently become secondary sources, e.g., a regular cotton shirt absorbed up to 3 mg of naphthalene when indirectly exposed to mothballs in a storage cabinet [81]. US EPA [62] notes that application rates are “imprecise,” ranging from 0.25 to 0.37 lbs per 12 ft^−3^ for mothballs or flakes used indoors as a moth repellant, and 1 lb per 12 ft^−3^ for flakes used indoors as an animal repellant.

More generally, naphthalene is used as a fumigant to repel animals and insects in closets, attics, soils (including gardens), and other applications, and also as a deodorizer in diaper pails and toilets. Outdoors, it is used to control nuisance vertebrate pests (snakes, squirrels, rats, rabbits, bats, *etc.*) around garden and building peripheries with an application rate from 0.56 to 10.8 lb of granules or flakes per treated area [62].

Many building materials emit naphthalene. A Canadian material emission database listed naphthalene in 41 of 69 commonly used materials [60]. On an area basis, caulking has the highest emission rate, 310 mg m^−2^ h^−1^, among materials tested, followed by carpet pads (installed underneath carpets), 2.1 to 9.9 mg m^−2^ h^−1^. Emission rates fell below 1 mg m^−2^ h^−1^ for other materials tested, which included solid and engineered materials and flooring materials. In a chamber study, naphthalene emissions from rubber floor covering with a loading ratio of 0.4 m^2^ m^−3^ and an air exchange rate (AER) of 0.5 h^−1^ resulted in a concentration of 3 μg m^−3^ [82]. Overall emission rates from materials in ten urban homes in Chicago, Illinois, estimated using AER measurements and steady-state and full-mixing assumptions, averaged 0.245 mg h^−1^, and the median was 0.115 mg h^−1^ [83].

#### 3.1.4. Emission inventories

A few countries have compiled estimates of nationwide emissions of naphthalene. Table 3 lists annual releases from the U.S., Canada, Netherlands, Scotland and Switzerland. Of these countries, the U.S. has the highest emissions: releases peaked at about 3,000 metric tons yr^−1^ in 1988 and 1998; more recent figures are in the 1,500 metric ton yr^−1^ range. The main source is the chemical industry, including the production of phthalic anhydride, and the manufacture of phthalate plasticizers, resins, dyes and insect repellents [2]. The chemical industry accounted for as much as 41% of (industrial) naphthalene emissions in 2006. The next largest category was the primary metals industry, which accounted for 28% of 2007 emissions. The data in Table 3 suggest downward trends over the last two decades in aggregate emissions in the U.S. and the Netherlands as well as U.S. mobile emissions; the trend for aggregate emissions in Canada appears to be flat. Decreases in aggregate emissions may be reflected in the declining trend of outdoor concentrations (depicted later in Figure 4).

As noted, emission inventory estimates have many limitations and may not reflect true releases. The quality of inventories, including their accuracy, validation and completeness, remains an issue. In fact, most inventories do not include naphthalene, instead focusing on criteria air pollutants and selected VOCs [84]. Additionally, inventories emphasize industrial and mobile sources, and most or all residential and commercial sources are generally excluded. While individually small, collectively these sources are important. For example, in 1989, about 5,500 metric tons of naphthalene were used as a moth repellent, 7,000 metric tons in 1994 [2], and 3,400 metric tons in 2008 [62]; essentially all of which was emitted to air. These quantities considerably exceed values listed in the national emission inventory (2,215 and 1,624 metric tons in 1989 and 1994, respectively). These values are uncertain, and they do not include off-label uses of naphthalene as a fumigant.

### 3.2. Exposure concentrations

This section reviews measurements of concentrations reported in indoor and outdoor studies. We also discuss key determinants of exposures identified in the literature.

In the atmosphere, naphthalene exists in both vapor and particulate phases. The distribution between these phases depends on temperature, precipitation and other environmental factors, but most naphthalene (>95%) occurs in the vapor phase [91,92,93,94]. Naphthalene’s atmospheric lifetime is short, less than 1 day [2]. The dominant transformation process is reaction with photochemically-produced hydroxyl and nitrate radicals, which produces nitro-naphthalene as a major product [95]. Rates of these reactions, and the atmospheric lifetime, will depend on ambient temperatures, the amount of sunlight, and the mix and concentration of other atmospheric constituents.

#### 3.2.1. Previous Reviews

Naphthalene concentrations in residences, along with other VOCs, have been reviewed in several papers. An older U.S. EPA report [96] summarized indoor air concentrations measured from 1987 to 1989 and, interestingly, listed naphthalene separately as a VOC, PAH and pesticide. As a VOC, the reported mean and median concentrations in 230 German homes were 2.3 and 2.0 μg m^−3^, respectively; as a PAH, concentrations were 1.0 and 2.2 μg m^−3^ in homes with and without smokers, respectively; and as a pesticide, concentrations ranged from 0.55 to 4.2 μg m^−3^ in U.S. residences. Brown *et al.* [37] reviewed 50 studies between 1978 and 1990, and estimated that the weighted average GM concentration was below 1 μg m^−3^ in various non-occupational indoor environments. Holcomb and Seabrook [97] listed average VOC concentrations in various microenvironments from 27 studies; naphthalene concentrations were reported in only two studies conducted before 1990 with quite high concentrations, from 9.2 to 13.9 μg m^−3^. Hodgson and Levin [98] estimated that the most representative median concentration in existing houses was 0.84 μg m^−3^. Wang *et al.* [99] reported naphthalene concentrations 0 to 1.8 μg m^−3^ in aircraft, but residential concentrations were not noted. Generally, naphthalene received little attention: it was omitted in other reviews of VOCs, e.g., Shah and Singh [100]; Dawson and McAlary [101], as well as in reviews of PAHs, e.g., Srogi [102] and Chang *et al.* [103].

Naphthalene-specific reviews have focused on its toxicity and health effects, although some have reported indoor and outdoor concentrations. The Report on Carcinogens [16] is limited to concentrations in workplaces. The IARC monograph [14] gives references only prior to 1995. The ATSDR toxicological profile [2], updated in 2003, includes references up to 1999, but omits many articles (which we cite in this review). Pruess *et al.* [11] reviews indoor and outdoor exposures, and proposes a hygiene-based exposure limit of 1,500 μg m^−3^, which is far above environmental limits. This review lists the means and ranges of concentrations found in 10 indoor residential studies and 21 outdoor studies. A recent summary of naphthalene exposure data [5] used a single study conducted in day care centers to represent indoor levels.

#### 3.2.2. Indoor concentrations

Table 4 summarizes 21 studies that measured indoor concentrations of naphthalene from 1986 to 2006. Concentrations are highly skewed, both within and between studies, as shown by study averages that greatly exceed medians, and by high peak concentrations (up to 144 μg m^−3^). Median concentrations ranged from 0.17 to 4.1 μg m^−3^, and averages from 0.8 to 9.5 μg m^−3^. Two studies showed strikingly high concentrations: the 150-home study in Syracuse, NY in which over 80% of the homes had one or more tobacco smokers indoors [104]; and a study of 27 residences which included many receiving complaints from occupants [105]. After excluding these studies, median concentrations ranged from 0.18 to 1.7 μg m^−3^ in non-smoking residences, which we present as a representative range for residences. Exaggerated by extreme values, means showed a wider range, 0.27 to 4.1 μg m^−3^. These ranges resemble those suggested in the previous reviews [37,96,98], and they encompass the value (0.95 μg m^−3^) used to estimate chronic inhalational doses and cancer risks [16].

Maximum concentrations reported in the different studies vary considerably, from around 1 to 144 μg m^−3^ (Table 4). The highest concentration (144 μg m^−3^) was observed in a home in Ottawa, Canada [113]; a nearly comparable level (92 μg m^−3^) was measured in a southeast Michigan, USA home [107]. However, most extrema were below 50 μg m^−3^, in accordance with Preuss *et al.* [11] who stated that “the majority of investigations found naphthalene levels far below 50 μg m^−3^.” We do not know whether these high concentrations reflect long-term levels and chronic exposure, or short-term excursions that might have occurred due to intermittent use of a naphthalene product or for other some other reason.

Although the effect of tobacco smoking is not always consistent (see below), we separately estimated concentrations in residences containing smokers. Concentrations averaged 1.8 μg m^−3^ in 10 Michigan homes [44], 9.5 μg m^−3^ in over 120 New York State homes [104], and from 1.3 to 2.5 μg·m^−3^ in 6 Ohio homes [111]. One median concentration was available, 2.8 μg m^−3^, in Syracuse, NY [104]. We propose a representative range of 1.8 to 9.5 μg m^−3^ for average (medians not available) naphthalene concentrations in residences with smokers, considerably higher than that just discussed for smoke-free residences.

Very elevated concentrations of naphthalene have been reported in rural homes in developing countries, such as China and African countries, where coal, wood and crop residues are widely used in simple and unvented cook stoves. For example, naphthalene concentrations averaged 28.7 μg m^−3^ in rural houses in Burundi [124]. While beyond our present scope, these studies suggest the need for further investigation given their significance and the scarcity of the data available.

**Figure 1.**
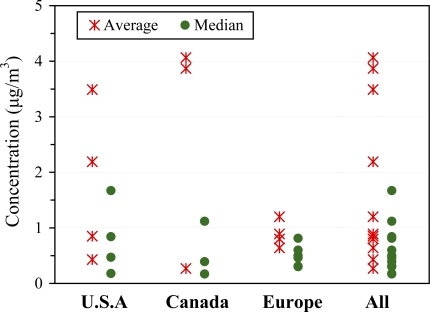
Indoor concentrations grouped by region.

Regional differences. Figure 1 displays ranges of median and mean concentrations for the U.S., Canadian and European studies. Naphthalene concentrations in US and Canada residences are similar. The North American studies show the widest range of concentrations, and levels generally exceed those in European studies, which show median concentrations below 0.6 μg m^−3^.

Urban *vs.* rural areas. Indoor concentrations did not show significant differences between rural and urban residences in one study [6]. Other (inter-study) comparisons also suggest little if any difference, e.g., the median concentration in a rural area in Missoula, MT, USA was only 0.3 μg m^−3^ [106], while similar or lower concentrations have been found in urban settings, e.g., 0.18 μg m^−3^ in the Chicago area [109]. Thus, excluding the measurements in rural areas [6,106] from the derivation of representative concentration ranges does not significantly affect the indoor concentration ranges.

**Figure 2.**
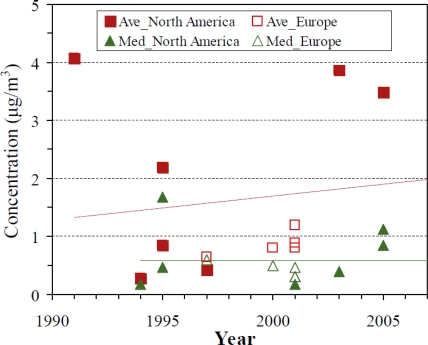
Long-term trends of naphthalene concentrations in residences. Ave = Average; Med = Median. Trend lines show indoor average: y = 0.04x – 81.27, R^2^ = 0.01; indoor median: y = −0.00x + 2.30, R^2^ = 0.00. The regression lines suggest essentially a flat trend and should not be used for quantitative predictions.

Long-term trends. Figure 2 shows study averages and median concentrations by year. Indoor levels did not show any clear trend, even after removing observations from smoking and complaint residences, or after stratifying by region or country. This differs from the decreasing trend seen for many indoor VOCs in North America [98] and Europe [118], and it is somewhat surprising given naphthalene’s decreasing share of the moth repellent market. The flat trend may hint that the older studies did not fully characterize naphthalene levels, possibly due to measurement problems as discussed later.

Seasonal variation. Indoor concentrations of naphthalene did not show consistent seasonal patterns. Higher concentrations were expected in winter due to lower air exchange rates and increased emissions from wood-burning fireplaces, which might explain trends seen in the nationwide Canadian study where concentrations averaged 6.7, 4.3 and 2.5 μg m^−3^ for outdoor temperatures ≤0 °C, 0–15 °C and ≥15 °C, respectively [115]. However, the opposite trend may occur due to higher emission rates from materials and outdoor barbecuing in summer, possible reasons for the higher levels in summer seen in ten Chicago area homes [109]. Seasonal cycles of aromatic VOCs (but not naphthalene) have been modeled in two German studies for aromatics [117,118].

#### 3.2.3. Determinants of indoor concentrations

Our review indicates that several emission sources influence indoor concentrations, including indoor tobacco smoking, use of moth repellents, and the presence of an attached garage. Additional factors affecting indoor concentrations include season, building characteristics (location, house age, and type of heating system), furniture, and ventilation condition. As discussed below, however, the evidence for these sources and factors is inconsistent, and few studies have quantitatively apportioned sources or the effects of these factors.

Attached garages. Garages containing vehicles or gasoline are known sources of aromatic compounds and naphthalene, although naphthalene concentrations in garages have been rarely reported [125]. Thus, garages attached to residences can significantly elevate indoor concentrations of aromatic compounds, including naphthalene [108,112]. In southeast Michigan homes with attached garages, sources in the garage were responsible for 35% of the indoor naphthalene levels; most of the remainder (65%) arose from indoor sources; and contributions from outdoor sources were negligible [126].

Indoor tobacco smoking. Elevated naphthalene concentrations were found in residences with tobacco smoking in an early study [111]. However, this was not the case in more recent studies, including studies that specifically evaluated effects of environmental tobacco smoke (ETS) [6,108,112], probably because ETS contributes only a small amount of naphthalene. Based on an analysis using ETS tracers, Charles *et al.* [44] estimated only about 3% of naphthalene was due to ETS. Nazaroff and Singer [10] provide a comparable estimate, a concentration increase of 0.1 to 0.2 μg m^−3^ due to smoking. These small contributions likely explain the inconsistent effect seen for indoor smoking.

Moth repellents. Elevated naphthalene concentrations due to indoor storage of mothballs were seen in a study of 10 residences [110]. Several older studies reported high indoor concentrations, 520 to 1200 μg m^−3^, in bedrooms and living rooms adjacent to closed spaces containing mothballs [23]. Price and Jayjock [5] provide some simple calculations of naphthalene concentrations in homes using mothballs, but supporting measurements are not given. Based on a mothball emission rate of 0.175 mg g^−1^ h^−1^, a ball or cake weight of 32 g [80], the typical U.S. house volume of 369 m^3^ and exchange rate 0.63 h^−1^, and a fully mixed (box) model [5], the indoor concentration of naphthalene is 24 μg·m^−3^. Concentrations would gradually decline, but the product would last about 8 months. Most users probably place a few mothballs in closed closets, plastic clothes bags, clothes chests or drawers about once per year. Such environments have limited exchange to the rest of the home, and emission rates can be affected by mass-transfer limitations and source-sink effects. These processes would tend to lower concentrations, but concentrations could be predicted using more complex, multicompartment models. Presently, *p*-dichlorobenzene appears to be the predominant ingredient in moth repellents sold in the U.S., however, naphthalene’s use in this application also continues. In many other countries, naphthalene continues to find more extensive use as both a repellent and deodorizer.

Griego *et al.* [20] state that Recochem, a Canadian company, is the sole U.S. registrant for pesticide use of naphthalene, but that off-label uses of mothballs as area fumigants, e.g., many mothballs placed on open trays in attics or other portions of homes, can elevated indoor levels by 10 to 300 μg m^−3^. The maximum indoor levels noted earlier, 90 and 144 μg m^−3^ in Michigan and Ottawa homes, respectively, fall in the range suggested for off-label uses, however, the specific use of naphthalene in these homes is unknown. Overall, historical exposures that resulted from the use of mothballs as well as off-label uses have not been documented.

Other factors. Domestic wood burning for heating has been reported to increase indoor PAH concentrations [127]. However, no significant difference was found in the single study that specifically examined fuel combustion and naphthalene levels [111]. Incense burning has been found to significantly increase indoor naphthalene concentrations in Asian homes [128].

Air fresheners have been stated as naphthalene sources [2], but Heroux *et al.* [112] found lower naphthalene concentrations in rooms containing air fresheners. Other chemicals appear to have replaced naphthalene use in this application, at least in the U.S.

Naphthalene concentrations have been positively related to home age [109], the opposite of what has been observed for other VOCs [105]. Lower naphthalene concentrations were found in homes with new furniture (≤12 months old), attributed to the sink effect [112].

In summary, few determinants of naphthalene concentrations that are consistent across the studies have been established. This may be caused by several factors. First, observed naphthalene concentrations are frequently low, often near or below method detection limits (MDLs). Measurements at such levels are prone to large uncertainties that may mask the true variation. Second, as discussed later, current monitoring methods for environmental naphthalene (as opposed to occupational settings) have not been fully validated. Third, the number of studies that have measured naphthalene is small, and most were not designed to identify determinants. Fourth, source-sink effects for naphthalene may be significant, and can complicate the collection of representative samples and the identification of primary (not re-emitting) sources. Lastly, in many cases there are multiple sources of naphthalene and source attribution is not always easy.

#### 3.2.4. Outdoor concentrations

Although the bulk of emissions occur to outdoor air, naphthalene is found at low concentrations in most urban and suburban settings. The 24 studies reviewed reported average outdoor concentrations below 1.0 μg m^−3^ and medians below 0.5 μg m^−3^. A set of studies considered to be representative of urban or suburban levels was developed by excluding the studies conducted in remote or rural areas, or those that used only one sampling site. This left 13 studies. Acknowledging some data quality issues, our estimate of representative urban and suburban concentrations are 0.02 to 0.31 μg m^−3^ for medians, and 0.01 to 0.82 μg m^−3^ for averages (Table 5). This range is within the 0.001 to 1.0 μg m^−3^ brackets suggested in a recent review [5].

Maximum outdoor concentrations ranged from 0.01 to 4.7 μg m^−3^ (Table 5). The upper bound concentration, about 5 μg m^−3^, might be used as a conservative exposure scenario for “general” urban air. Occasionally, higher concentrations have been detected, e.g., 25 μg m^−3^ was observed outside of eight Hangzhou, China homes [123], but this may have resulted from entraining indoor air. Levels may be elevated near industrial and waste disposal sites containing strong sources, e.g., naphthalene levels averaged 11 μg m^−3^ at a landfill site in summer in Guangzhou, South China [129].

**Figure 3.**
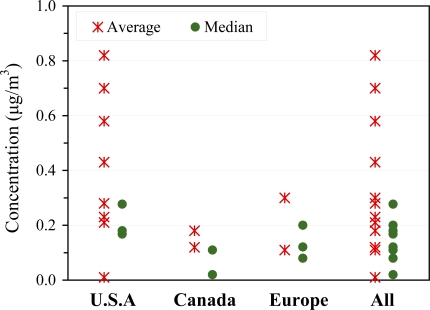
Outdoor concentrations grouped by region.

Regional differences. Median outdoor concentrations of naphthalene in urban areas ranked by region generally follow the following trend: U.S.A. > Europe > Canada (Figure 3). Median concentrations measured elsewhere (as those summarized in Table 5) fall within the bounds of these studies. It is not feasible to compare ambient concentrations measured in rural or remote areas due to the very low concentrations seen and the small number of studies.

Temporal variation. Like many other pollutants, naphthalene concentrations in outdoor air undergo both diurnal variation. Concentrations tend to peak at night and in the morning, and are lowest at mid-day [40,137]. Nighttime increases are attributable to low mixing heights that build up levels from local sources, while morning peaks are associated with vehicle emissions at rush-hour and diminished dispersion. Levels decrease during midday due to enhanced dispersion, lower traffic and reduced emissions. A simulation study predicted peak concentrations increasing by roughly 0.2 μg m^−3^ due to weak vertical mixing and photo-oxidation [40]. This pattern is similar to that seen for traffic-related VOCs such as benzene, toluene and 1,3-butadiene [138] and some PAHs [139].

Outdoor concentrations also show seasonal variations, and higher levels are seen in winter as compared to summer [40,130,131,137]. Seasonal changes were 0.96 μg m^−3^ in Los Angles and 0.20μg·m^−3^ in Riverside, California, USA [131]. (Other studies did not quantify seasonal changes.) In general, the seasonal changes follow patterns seen for VOCs, *i.e.,* in winter, cool temperatures are often associated with decreased photochemical reaction rates [140], increased emissions from heating sources [39], and decreased dispersion due to more stable air and lower mixing heights [141].

**Figure 4.**
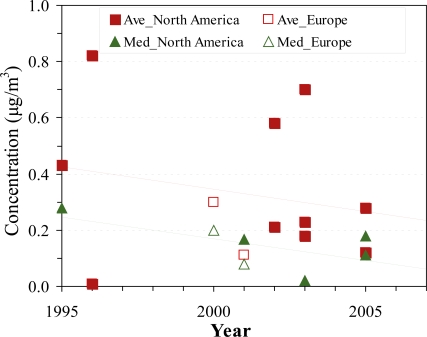
Long-term trends of naphthalene concentrations in residences. Ave = Average; Med = Median. Trend lines show outdoor averags: y = −0.02x + 31.78, R^2^ = 0.05; and outdoor median: y = −0.02x + 31.03, R^2^ = 0.39. The regression lines suggest a decreasing trend but should not be used for quantitative predictions.

All but one of the ambient studies used sampling periods less than 3 years in length, and thus long-term trends were not investigated. In Leipzig, German, ambient measurements were collected for 8 years, but naphthalene trends were not identified [118]. We attempted to discern trends by aggregating the 12 urban studies, and plotting means and medians against the 15 years spanned (Figure 4). These data suggest a downward trend, especially for median concentrations, but the variation is very large across the studies. Such multi-study comparisons cannot identify trends occurring at specific sites, which are best examined using long-term monitoring at the same sites, consistent methods, and sampling strategies that account for diurnal and seasonal variation.

Rural areas. Unsurprisingly, outdoor concentrations are lower in rural areas that have little traffic and fewer if any industrial facilities (Table 5). Based on measurements in Missoula, MT, U.S. [106] and rural Western Canada [134], rural concentrations typically fall below 0.1 μg m^−3^. Several studies have compared urban and rural areas. In North Carolina, U.S., the average concentration at rural sites was four times lower than at inner city sites in Durham [6]. In southern California, average naphthalene concentrations ranged from 0.06 to 0.27 μg m^−3^ in two rural communities [91]. Occasionally, rural areas experience locally elevated concentrations due to open burning of crop residues and possibly other materials, as shown in an agricultural county in Taiwan where naphthalene levels increased by 1.3 to 2.6 times to 0.38 to 0.44 μg m^−3^ during the burning period [93]. Such events are sporadic but may result in air pollution episodes with unfavorable meteorology [142,143]. Our search failed to find any measurements of naphthalene during air pollution episodes.

Remote locations. Naphthalene concentrations measured at remote locations, such as rural areas of Western Canada, typically range from below method detection limits (<MDLs) to 0.01 μg m^−3^ [134]. This is similar to the 0.0001 to 0.003 μg m^−3^ range suggested by Price and Jayjock [5]. These “background” naphthalene concentrations are small and generally negligible compared to urban levels.

#### 3.2.5. Determinants of outdoor concentrations

Traffic. Although a known constituent of vehicle exhaust, few studies have examined the effect of traffic on naphthalene concentrations. Naphthalene has been rarely reported in tunnel studies, a common way to characterize traffic emissions. One study in two highway tunnels in Chicago reported a concentration of 8.0 μg m^−3^ [144]. In a multi-community monitoring program in Southern California, naphthalene concentrations varied from 0.06 μg m^−3^ in a community with light traffic to 0.58 μg m^−3^ in a community traversed by 200,000 vehicles day^−1^ [91]. The naphthalene gradient due to traffic-related emissions has not been characterized.

Proximity to industrial facilities can increase naphthalene concentrations. In Sarnia, Canada, a clear concentration gradient was seen around a large cluster of industrial and chemical facilities [133]. In southeast Michigan, higher concentrations were observed in an industrial city than a suburban community in Michigan [107]. A modeling study of Los Angeles showed a similar pattern [40]. As mentioned earlier, quite high concentrations (11 μg m^−3^) were noted at a landfill site in China in summer [129]. A slightly elevated concentration, 1.1 μg m^3^, was seen near a former manufactured gas plant [145]. (Note that this concentration was incorrectly reported (as 1,100 μg m^3^) in this paper due to a unit problem.)

In summary, ambient concentrations of naphthalene vary spatially and temporally. Concentrations in urban areas typically range from 0.02 to 0.31 μg m^−3^; levels are much lower in rural and remote areas, below 0.1 μg m^−3^. Particularly in urban areas, concentrations show diurnal and seasonal patterns, reflecting variability in emission sources and meteorological influences. Rural areas may experience occasional elevated concentrations due to open biomass burning. While several studies used a large number of measurements, no long-term trends were discerned, and this was not investigated in any of the studies. Outdoor concentrations form a “floor” for indoor concentrations, *i.e.,* they represent the level in residences that do not contain naphthalene-containing products.

#### 3.2.6. Personal exposures

Personal exposure to naphthalene have been reported in only three European studies (Table 6). These show median concentrations from 0.5 to 2.0 μg m^−3^ and averages from 0.78 to 2.3 μg m^−3^. These levels are within the ranges discussed for indoor residential concentrations (and much higher than outdoor concentrations), suggesting that personal exposures mainly result from exposures and sources indoors. The maximum personal exposures varied from 2.7 to 12.7 μg m^−3^. The maxima ranked as urban > suburban > rural in the U.K.; median and mean concentrations did not depend on urbanization [146]. Edwards *et al.* [121] made simultaneous indoor, outdoor and personal measurements, and found that personal exposures (including means, medians, and maxima) were lower than indoor concentrations. This suggests that personal contact with naphthalene sources is rare, and that the “personal cloud” effect seen for some other pollutants may not apply to naphthalene. This seems reasonable given that individuals spend minimal amounts of time in a closet or bathroom where mothballs, deodorizers and air fresheners might be used, while monitoring in nearby rooms might end up with high concentrations, and since individuals are outdoors or in other environments where levels are low.

A recent UK study [146] thoroughly analyzed determinants of personal exposure, and found that the presence of an attached garage and exposure to ETS were determinants of VOC exposures, and ETS was the primary determinant of PAH exposures. However, these factors did not significantly affect naphthalene exposures. Other factors, including community setting and property value, also were not influential.

The lack of studies examining personal exposures in North America, where naphthalene has found extensive indoor use and where both indoor and outdoor concentrations are higher, represents a significant gap in the literature. While largely beyond our scope, the same gap appears to exist in the occupational setting, e.g., Rappaport *et al.* [29] used exclusively European studies to establish the relationship between personal naphthalene exposure and total PAH exposures. Information is also lacking for Australia and other countries where indoor use of naphthalene is common.

### 3.3. Health risk assessment

As noted at the onset, health risk assessments for naphthalene are in flux. The current reference concentration (RfC) of 3 μg m^−3^ established by US EPA [17] represents a threshold effects level. Five studies have reported higher average indoor concentrations: one study of mostly smoking homes [104]; one including homes receiving occupant complaints [105]; and three more representative studies [107,113,115]. None of the outdoor studies and none of the median concentrations in the indoor studies exceeded the RfC.

The estimated cancer risks from naphthalene exposure are notable. The 2002 National-Scale Assessment concluded that naphthalene in ambient air was a regional cancer risk “driver”, defined as an air toxic where the typical individual chronic cancer risk exceeded 10^−5^ [21]. Naphthalene was ranked the second highest indoor risk driver and the third highest outdoor risk driver in a Michigan study [107]. Loh *et al.* [36] derived personal exposures using a microenvironmental model, and ranked naphthalene as ninth among 19 carcinogenic air pollutants. Using the draft URE [18], cancer risks will increase 3-fold and typical risk levels will approach or fall into the 10^−4^ range, while peak measurements, if reflective of chronic exposure, represent cancer risks in the 10^−3^ or possibly even higher range. If the tumor response in experimental animals is confirmed to be predictive of human health risk, these risks will be significant, especially given the millions of individuals exposed.

The true cancer risk due to naphthalene exposure remains controversial. Much of the debate focuses on site concordance, that is, whether carcinogenic effects seen in experimental animals [3] can be extrapolated to humans. Both U.S. EPA [18] and IARC [14] judge that epidemiological data are inadequate for determining human carcinogenicity. In a screening level assessment, the predicted number of naphthalene-induced nasal tumor cases in the U.S. using the draft EPA unit risk factor (URFs) was 65,905, far exceeding the 910 observed [23]. Clearly, the URFs derived from animal data have large uncertainties.

## 4. Discussion

Naphthalene is one of the least volatile VOCs and the most volatile PAH. Because this compound has often been excluded in both VOC and PAH studies, the exposure-relevant literature is, in many ways, deficient and inferior to the VOC and PAH literature. Still, we identified 20 recent indoor studies and 21 recent outdoor studies, which were used to derive representative ranges of concentrations applicable to residences and urban settings. Only three studies making personal measurements in community settings were identified, all in Europe.

### 4.1. Information gaps

We note a number of important information gaps. For indoor settings, the available studies are suggestive but inconsistent with respect to the influence of potential naphthalene sources, such as moth repellents, air fresheners, and deodorizers. There is little if any quantification of these sources. In the outdoor studies, information regarding emission inventories, long range transport, source apportionments and long-term trends is incomplete, certainly as compared to other VOCs and PAHs [148]. Large gaps exist regarding the availability of information on personal exposures, and no North American studies were identified. This is an important gap since personal exposure measurements are considered the best estimate of true exposures [149] and since U.S. and Canadian homes tended to have higher indoor concentrations than European homes. We did note that sample sizes were often limited. Finally, the suggested representative ranges for outdoor, indoor and personal measurements are rather large. We have limited confidence in the upper range of concentration measurements, e.g., our recent work has shown several homes in Detroit with naphthalene concentrations far above any listed in Table 4 (unpublished data).

Comparisons among different studies are complicated by sampling issues (discussed below) and different siting criteria used in the various studies. Clearly, it is important to differentiate studies using monitoring sites designed to reflect urban population exposure from those designed to capture “hotspots” due to industry or traffic, as well as studies intended to characterize “background” and “remote” conditions.

### 4.2. Measurement issues

Naphthalene has not been included in many VOC studies, in part due to limitations of the sampling techniques. Whole-air canister sampling (TO-15), a standard U.S. EPA method [150], does not include naphthalene as a target compound, and canister methods for this compound have not been validated. Thus, ambient concentrations of naphthalene are not monitored in the nationwide Urban Air Toxics Monitoring Program. The standard adsorbent-based methods (TO-17), using either active (pumped) or passive (diffusion) sampling [151], also do not specify naphthalene as a target compound. While used, the method has been only partially validated [152]. Two popular and commercially available passive adsorbent samplers, OVM and Radiello, were not intended for naphthalene in their initial design [153,154].

None of the studies reviewed had much if any discussion of data quality issues, e.g., blank contamination, reproducibility and detection limits. Our evaluations using Tenax GR and Carbosieve adsorbents, short-path thermal desorption, and GC/MS analysis show reasonable performance can be obtained using adsorbent-based methods, although the recovery, reproducibility and other performance indicators for naphthalene are often inferior to that for other VOCs [155].

In outdoor air, vapor phase PAHs are frequently collected using high volume methods, typically with polyurethane foam (PUF) adsorbents and flow rates of 255 L min^−1^ (TO-13A) [156]. This method was not recommended for naphthalene due to low recovery efficiency, low storage capability [156] and high breakthrough [156,157]. Because naphthalene is typically present at concentrations that are one to two orders of magnitude higher than other PAHs, it is sometimes excluded from chemical and data analyses. Price and Jayjock [5] have suggested that naphthalene seems to be included either as a VOC or PAH for sake of completeness but that the collected data were not thoroughly analyzed.

We did not locate published reports that systematically documented laboratory or field inter-comparisons between canister or adsorbent-based sampling for VOCs, and PUF sampling for PAHs. A recent case study shows that EPA TO-15 generally yields higher concentrations for vapor phase naphthalene than EPA TO-13A [158]. In indoor applications, we have shown reasonable agreement (within factor of two) between VOC sampling using passive methods and Tenax GR adsorbents and active medium flow PUF sampling (unpublished data). However, comprehensive and robust performance evaluations are needed. While consistent and low MDLs are ideal for exposure assessment [159], the various measurement techniques attain very different MDLs, e.g., standard VOC measurements attain MDLs of about 0.01 μg m^−3^ [160], while PUF methods can go orders of magnitude lower.

## 5. Conclusion

Concern regarding human exposure to naphthalene through inhalation has greatly increased due to its potential carcinogenicity, which was discovered in 2000 [3]. We derived representative ranges of residential, outdoor and personal concentrations of naphthalene, emphasiz ing the more recent literature. This literature is limited, especially for personal exposures. Considering what is available, we conclude that personal and residential concentrations are similar, while ambient concentrations are about an order of magnitude lower. Our estimate of representative ranges of indoor concentrations are about 0.2 to 2 μg m^−3^ for medians, and about twice that for averages.

We did not observe a decline in indoor concentrations over the past 10 to 15 years, in contrast to trends seen for other VOCs and PAHs, however, outdoor measurements did appear to decline. We anticipate that decreased indoor smoking, improved emission controls on vehicles, and substitution of the naphthalene in moth and other animal repellents and deodorizers has significantly reduced exposures in the U.S. However, available data are not ideal for quantitative trend studies.

Most measurements fall below the current U.S. EPA reference concentration of 3 μg m^−3^ established for non-cancer effects, although measurements in several homes show concentrations approaching or exceeding 100 μg m^−3^. Outdoor exposures, except where there are strong industrial sources, are well below the RfC. However, using the available cancer risk factors, some of which are draft and under review, indoor and outdoor concentrations correspond to individual risks in the 10^−5^ to 10^−3^ range, very high for an environmental exposure. The cancer risk factors have large uncertainties and are controversial, but in many assessments naphthalene ranks at or near the top of those substances posing inhalation cancer risks. This analysis suggests that further study, control and abatement are warranted. We anticipate much higher exposures and risks in countries where these controls are lacking, or where other sources are present.

We noted a number of important information gaps and research needs. Existing exposure data are limited, and monitoring surveillance should be improved. There is a need to validate and intercompare VOC and PAH measurement techniques. This will also ensure the comparability of studies and reduce uncertainties. The spatial and temporal variability of concentrations near roads, industrial and other sources, is poorly characterized. Factors affecting indoor concentrations, including the causes of the highest levels, are not well understood, and populations at risk of high of exposure presently cannot be identified. Better information regarding product usage patterns, emission rates of consumer products, building materials, and other sources of naphthalene is needed, as are long term measurements. Future studies might address losses due to adsorption onto building materials, chemical reactions, and utilize multicompartment models to better understand current and estimate historical exposures.

## Figures and Tables

**Table 1.** Exposure limits and guidelines for naphthalene.

**Organization**	**Reference Level**	**Unit**	**Interpretation**	**Year**	**Ref**
**Environmental**					
Environmental Protection Agency (EPA)	3	μg m^−3^	Inhalation RfC	1998	[17]
Agency for Toxic Substances and Disease Registry (ATSDR)	3.6	μg m^−3^	Inhalation MRL (Chronic)	2005	[30]
Office of Environmental Health Hazard Assessment (OEHHA), California	9	μg m^−3^	Inhalation REL (Chronic)	2000	[31]
Office of Environmental Health Hazard Assessment (OEHHA), California	3.4 × 10^−5^	per μg m^−3^	Inhalation Unit Risk	2009	[22]
Michigan Department of Environmental Quality (MDEQ)	3 0.08 0.8	μg m^−3^	ITSL (24 hr) IRSL (cancer risk of 10^−6^) SRSL (cancer risk of 10^−5^)	2004	[32]
**Occupational**					
Occupational Safety & Health Administration (OSHA)	50	μg m^−3^	PEL (TWA)	2001	[33]
National Institute for Occupational Safety and Health (NIOSH)	50 75	μg m^−3^	REL (TWA) REL (STEL, 15 min)	2005	[34]
American Conference of Governmental Industrial Hygienists (ACGIH)	50 75	μg m^−3^	TLV (TWA) TLV (STEL, 15 min)	2009	[35]

Notes: RfC = Reference Concentration; MRL = Minimal Risk Level; REL = Reference Exposure Level; ITSL = Initial Threshold Screening Level; IRSL and SRSL = Initial and Secondary Risk Screening Levels; PEL = Permissible Exposure Limit; REL = Recommended Exposure Limit; TLV = Threshold Limit Value; TWA = Time Weighted Average; STEL = Short Term Exposure Limit. This Table does not include the lower levels given by EPA [18] in a “do not cite or quote” draft document discussed in the text. Ref = Reference.

**Table 2.** Emission factors for naphthalene and selected sources.

**Emission source**	**Emission factor**	**Unit**	**Ref**
**Industrial stacks, furnaces, and boilers**			
Industrial stacks	69–2707	μg/kg	[42]

Fueled-boilers	10900	μg/kg	[43]
Diesel fueled-boiler	1263	μg/kg	
HO-NG fueled-boiler	1835	μg/kg	
COG-BFG fueled-boiler	37.3	μg/kg	

Joss paper furnaces	41.2	mg/kg	[46]
**Combustion of wood and coals**			
House coal	19	mg/kg	[48]
Hardwood	8.2	mg/kg	
Pine wood	4–27.67	mg/kg	[49]
Rice husk briquettes	18.06	mg/kg	
Anthracite coal	Nd	mg/kg	

Birchwood	52.8	mg/kg	[51]
Pinewood	71.4	mg/kg	
Wood waste	9.1	mg/kg	
Peat briquette	71.4	mg/kg	
Domestic Waste	331.5	mg/kg	

Pine	227	mg/kg	[52]

Wood	39.1	mg/kg	[54]
Coal briquette	44.5	mg/kg	
Charcoal	7.48	mg/kg	

Almond	7.3	mg/kg	[41]
Walnut	14.6	mg/kg	
Fir	13.6	mg/kg	
Pine	17.0	mg/kg	
**Burning of agricultural residue**			
Rice straw	5.0–5.7*	mg/kg	[58]
Bean straw	1.8–3.6*	mg/kg	

Agricultural debris	25.2	mg/kg	[59]

Barley	11.1–149.5	mg/kg	[41]
Corn	1.3–7.6	mg/kg	
Rice	7.3–9.6	mg/kg	[41]
Wheat	44.4–348	mg/kg	

Tobacco smoke^**^			
Commercial cigarette	13.2	μg/ciga	[44]

Research cigarette	15.1–18.1	μg/ciga	[45]

In wallboard only room	26–54	μg/ciga	[47]
In wallboard/carpet room	28–42	μg/ciga	
In fully furnished room	17–34	μg/ciga	
**Mobile**			
Catalyst-equipped gasoline-powered vehicle	1	mg/km	[50]
Non-catalyst-equipped gasoline-powered vehicle	50	mg/km	

Heavy-duty diesel vehicles-Idle	10.2	μg/mile	[53]
Heavy-duty diesel vehicles-Creep	505	μg/mile	
Heavy-duty diesel vehicles-Transient	276	μg/mile	
Heavy-duty diesel vehicles-Cruise	20.1	μg/mile	

Helicopter	503	μg/m^3^	[55]

Ship auxiliary engine	72–5850	μg/kWh	[56]

Ship	6.5–244	μg/m^3^	[57]
**Household materials**			
Caulking	310.0	g/(m^2^h)	[60]
Adhesive	1	g/(m^2^h)	
Flooring materials	0.001–57.7	g/(m^2^h)	
Wood materials	0.02–0.2	g/(m^2^h)	

*: sum of the vapor and particulate phases. Ref = “Reference”.

**: as emitted in side-stream smoke.

**Table 3.** Annual releases of naphthalene in the U.S., Canada, The Netherlands, Scotland and Switzerland.

Year	US Industry (ton)	US Mobile (ton)	Canada (ton)	Netherlands (ton)	Scotland (kg)	Switzerland (kg)
2008		2,913		58	560	
2007	1,290		332	115	294	30
2006	1,521		504	115	19	
2005	1,755	3,761	656	118	18	
2004	1,560		294		35	
2003	1,646		190			
2002	1,368	5,151	358			
2001	1,205		168			
2000	1,400		221	133		
1999	1,747		253			
1998	2,729		201			
1997	1,504		613			
1996	1,837		100			
1995	1,510		69	196		
1994	1,624		113			
1993	1,470					
1992	2,299					
1991	1,831					
1990	2,286			263		
1989	2,215					
1988	3,049					

Reference	[85]	[86]	[87]	[88]	[89]	[90]

**Table 4.** Naphthalene concentrations measured in residences.

**Country**	**Location**	**Setting**	**Sampling period**	**No. of residences**	**Sampling method**	**DF**	**Concentration (μg m^−3^)**					**Rep**	**VOC/PAH**	**Ref**
							**AM**	**SD**	**GM**	**Median**	**Max**			
US	Missoula, MT	Rural	2005–2006	51 high school students’ homes	12-h active sorbent		-	-	-	0.3	1.4	N	VOC	[106]
US	Southeast MI	Urban and suburban	2004–2005	159 homes	4-d passive sorbent	100%	3.49	-	-	0.84	91.75	Y	VOC	[107, 108]
US	Syracuse, NY	Urban	2001–2003	150 residential buildings	24-h active sorbent		9.52	-	-	2.84	44.7	N	VOC	[104]
US	Chicago, IL	Urban and suburban	2000–2001	10 homes	48-h active PUF		-	-	-	0.18	2.34	Y	PAH	[109]
US	Raleigh-Durham-Chapel Hill Area, NC	Urban	1997	9 children’s homes	48-h active PUF		0.43	-	-	-	1.24	Y	PAH	[7]
US	Five cities, NC	Urban and rural	1995	24 low-income families	24-h active resin		2.19	1.87	-	*1.67*	9.7	Y	PAH	[6]
US	Southeast Chicago, IL	Urban	1994–1995	10 homes	24-h active PUF	89%	0.85	0.95	-	0.47	50	Y	PAH	[110]
US	Columbus, OH	Urban	1986–1987	8 homes	8-h active resin		1.4	-	-	-	4.2	N	PAH	[111]
Canada	Quebec City, Quebec	Urban	2005	96 dwellings	7-d passive sorbent	100%	-	-	1.45	1.12	23.02	Y	VOC	[112]
Canada	Ottawa, Ontario	Urban	2002–2003	75 residences	100-min active sorbent	83%	3.87	17.25	0.33	0.39	144.44	Y	VOC	[113]
Canada	Montreal, Quebec	Urban	1991–1994	18 residences	24-h active resin	100%	0.27	-	0.17	*0.17*	-	Y	PAH	[114]
Canada	Canada nationwide		1991	754 homes	24-h passive sorbent		4.07	-	-	-	-	Y	VOC	[115]
UK	Birmingham	Urban	1999–2000	12 homes	Active sorbent		0.8	1	-	0.5	6	Y	VOC	[116]
Germany	Leipzig, Munchen, and Koln	Urban	1994–2001	2103 measurements	4-week OVM passive		0.8	-	-	0.3	1.8	Y	VOC	[117]
Germany	Leipzig	Urban	1994–2001	222 measurements	4-week OVM passive		0.89	-	-	0.31	40.79	Y	VOC	[118]
Germany	Bremer	Urban	NA	182 measurements	Active PUP	100%		-	-	0.81	30.91	N	PAH	[119]
Germany	Schleswig-Holstein	Urban	2000–2001	39 dwellings and houses	Active sorbent		1.2	2.8	0.31	0.46	14	Y	VOC	[120]
Finland	Helsinki	Urban	1996–1997	201 homes	48-h active sorbent	24%	0.64	0.53	0.55	*0.6*	3.89	Y	VOC	[121]
Finland	NA	NA	NA	50 normal houses	Active sorbent		0.44	0.46	-	0.31	1.63	N	VOC	[122]
Australia	Melbourne	Urban	N/A	22 non-complaint homes 5 complaint homes	30–50 min active sorbent	30%	3.2 6.9	-	1.6 4.1	*1.6* *4.1*	-	N	VOC	[105]
China	Hangzhou	Urban	1999	8 nonsmoking and smoking homes	XAD-2 resin	100%	6.77	6.90	3.94	4.59	20.57	N	PAH	[123]

Notes: DF = “Detection frequency”; AM = “Arithmetic mean”; SD = “Standard deviation”; GM = “Geometric mean”; Max = “Maximum”; Rep = “Representativeness”; Y = “Yes”; N = “No”; VOC = “Volatile organic compound”; PAH = “Polycyclic aromatic hydrocarbon”. Medians in italics are derived using eq. (1). Ref = “Reference”.

**Table 5.** Naphthalene concentrations measured in ambient air. Otherwise as Table 4.

**Country**	**Location**	**Setting**	**Sampling period**	**No. of sampling locations**	**Sampling method**	**DF**	**Concentration (μg m^−3^)**					**Rep**	**VOC/PAH**	**Ref**
							**AM**	**SD**	**GM**	**Median**	**Max**			
US	Missoula, MT	Rural	2005–2006	Outside of 51 high school students’ homes	12-h active sorbent		-	-	-	0.1	0.4	N	VOC	[106]
US	Southeast Michigan	Urban and suburban	2004–2005	Outside of 159 homes	4-d passive sorbent	94%	0.28	-	-	0.18	4.72	Y	VOC	[107]
US	Chicago, IL	Urban and suburban	2000–2001	Outside of 10 homes	48-h active PUF		-	-	-	0.17	1.87	Y	PAH	[109]
US	Raleigh–Durham–Chapel Hill, NC	Urban	1997	4 sites	48-h active PUF		0.06	-	-	-	0.076	N	PAH	[7]
US	Five cities in NC	Urban and rural	1995	Outside of 24 low-income families	24-h active resin		0.43	0.51	-	*0.28*	1.82	Y	PAH	[6]
US	Columbus, OH	Urban	1986–1987	Outside of 8 homes	8-h active resin		0.17	-	-	-	0.33	N	PAH	[111]
US	Phoenix and Tucson, AZ	Urban	1994–1996	5 sites, 305 samples	6L canister		0.01–0.82	-	-	-	1.96	Y	VOC	[130]
US	San Dimas, Upland, Mira Loma, Riverside, CA	Urban	2001–2002	4 schools	24-h active PUF		0.21–0.58	-	-	-	1.04	Y	PAH	[91]
US	Los Angeles, CA Riverside, CA	Urban	2002–2003	2 sites	5-day active sorbent		0.7 0.23	-	-	-	2.54 0.77	Y	PAH	[131]
US	Wildlife Refuge, MS	Remote	1991	2 sites, 80 samples	4-day active PUF		0.0001	-	-	*-*	-	N	PAH	[132]
Canada	Sarnia, Ontario	Urban	2005	37 sites	2-week OVM passive		0.12	0.05	-	*0.11*	-	Y	VOC	[133]
Canada	Ottawa, Ontario	Urban	2002–2003	Outside of 74 homes	100-min active sorbent	54%	0.18	-	-	*0.02*	3.9	Y	VOC	[113]
Canada	Western Canada	Rural	2004	11399 samples	1-month OVM passive	70%	0.008	-	0.003	*0.003*	1.7	N	VOC	[134]
UK	Birmingham	Urban	1999–2000	Outside of 12 homes	Active sorbent		0.3	0.2	-	0.2	0.9	Y	VOC	[116]
UK	Birmingham	Urban	1992	1site, 55 samples	24-h active PUF		0.002–0.012	-	-	-		N	PAH	[92]
Germany	Leipzig	Urban	1994–2001	222 measurements	4-week OVM passive		0.1	-	-	0.1	1.5	Y	VOC	[118]
Germany	Germany	Urban	NA	47 measurements	Active PUP	100%	-	-	-	0.1	1.4	Y	PAH	[119]
Finland	Helsinki, Finland	Urban	1996–1997	Outside of 183 homes	48-h active sorbent	<20%	-	-	-	-	1.3	N	VOC	[121]
Australia	Melbourne	Urban	N/A	27 sites	30–50 min active sorbent	30%	<MDL	-	-	<MDL	-	N	VOC	[105]
India	Delhi	Urban	2001	Multiple sites	4-h active sorbent		0.39	0.30	-	*0.31*	-	Y	VOC	[135]
India	Mumbai	Urban	2001–2002	Multiple sites	4-h active sorbent		0.10	0.12	-	0.06	-	Y	VOC	[136]
Korea	Seoul	Urban	1999	1 site	24-h active PUF		0.01	0.01	-	*0.01*	-	Y	PAH	[137]
China	Hangzhou	Urban	1999	Outside of 8 homes	XAD-2 resin	100%	6.31	6.82	3.15	4.15	19.83	N	PAH	[123]
		Industrial					0.41	-	-	*-*	-	N		
Taiwan	Taichung	Urban	2002	1 site	3-day active PUF		0.28	-	-	*-*	-	Y	PAH	[94]
		Rural					0.22	-	-	*-*	-	N		

**Table 6.** Personal exposure measurements of naphthalene. Otherwise as Table 4.

**Country**	**Location**	**Setting**	**Sampling period**	**Sample size**	**Sampling method**	**DF**	**Concentration (μg m^−3^)**					**Rep**	**VOC/PAH**	**Ref**
							**AM**	**SD**	**GM**	**Median**	**Max**			
UK	London and Birmingham	Urban		191			0.78	1.49	0.49	*0.49*	12.67	Y		
	Birmingham	Suburban	2005–2007	209	5-day sorbent active		0.72	0.75	0.55	*0.55*	6.35	Y	VOC	[146]
	Midlands and Wales	Rural		100			0.71	0.54	0.58	*0.58*	2.84	N		
Germany	West Germany	Urban	1990–1991	113	7-day OVM passive	96%	2.3		2.1	2.0	4.0	Y	VOC	[147]
Finland	Helsinki	Urban	1996–1997	183	2-day sorbent active	10%	na		na	na	2.7	N	VOC	[121]
