# Correction: Jia, C. and Batterman, S. A Critical Review of Naphthalene Sources and Exposures Relevant to Indoor and Outdoor Air. *Int. J. Environ. Res. Public Health* 2010, 7, 2903–2939

**DOI:** 10.3390/ijerph8083191

**Published:** 2011-07-28

**Authors:** Chunrong Jia, Stuart Batterman

**Affiliations:** 1 School of Public Health, University of Memphis, 121 Browning Hall, Memphis, TN 38152 USA; E-Mail: cjia@memphis.edu; 2 Department of Environmental Health Sciences, University of Michigan, 1420 Washington Heights, Ann Arbor, MI 48109-2029 USA

The authors would like to make the following corrections to their published paper:

1. Table 1, p. 2906: units for all of the occupational standards (*i.e.*, units in the last three rows) should be in mg/m^3^; not in μg/m^3^.
2. Table 2, p. 2909: units for reference [60] should be in μg/(m^2^ h); not in g/(m^2^ h).
3. Paragraph 4, lines 3 and 4, p. 2911: For the following sentence citing reference [60], units should be in μg/(m^2^ h); not in mg/(m^2^ h).
  On an area basis, caulking has the highest emission rate, 310 μg m^−2^ h^−1^, among materials tested, followed by carpet pads (installed underneath carpets), 2.1 to 9.9 μg m^−2^ h^−1^. Emission rates fell below 1 μg m^−2^ h^−1^ for other materials tested, which included solid and engineered materials and flooring materials.

We apologize for any inconvenience caused to the readers.

